# Temporal stability of semantic predictions in subclinical autistic and schizotypal personality traits

**DOI:** 10.1038/s41537-025-00643-9

**Published:** 2025-07-19

**Authors:** Elisabeth F. Sterner, Andrea Greve, Franziska Knolle

**Affiliations:** 1 Department of Diagnostic and Interventional Neuroradiology, School of Medicine and Health, Technical University of Munich, Munich, Germany; 2 Department of Experimental Psychology, Ludwig-Maximilians University Munich, Munich, Germany; 3 Graduate School of Systemic Neurosciences (GSN), Ludwig-Maximilians University Munich, Munich, Germany; 4 Medical Research Council Cognition and Brain Sciences Unit, University of Cambridge, Cambridge, UK; 5 Department of Psychology, University of Cambridge, Cambridge, UK

**Keywords:** Schizophrenia, Psychosis

## Abstract

Language impairments are core symptoms of both schizophrenia and autism spectrum disorders and have been linked to deficits in predictive language processing. While altered use of semantic predictions have been reported in both conditions, little is known whether semantic predictions are stable over time. The goal of this study was therefore to investigate the temporal stability of semantic prior beliefs focusing on individuals with schizotypal and autistic traits. 115 participants, assessed for subclinical schizotypal (SPQ_5ls_; mean = 77.99, SD = 39.31) and autistic traits (AQ; mean = 15.67, SD = 6.01), completed an auditory stability paradigm at two timepoints to investigate the temporal stability of semantic predictions. At timepoint one, consisting of one session, participants listened to 240 sentence beginnings varying in predictability (e.g., high: “The swimmer jumped into the…”; low: “The child hid the toy under the…”) and provided a prediction for each sentence-final word. Timepoint two, consisting of two sessions, each session comprising of 120 old and 120 new sentences. In addition to final-word predictions, sentence recall was assessed to examine the influence of memory on prediction stability. Generalized linear mixed models revealed that higher predictability led to greater temporal stability of semantic predictions. Importantly, increasing schizotypal and autistic traits were associated with reduced stability, particularly in highly predictable contexts where stable predictions typically facilitate efficient language processing. While poorer sentence recall was linked to greater instability, especially in medium- and low-predictability contexts, it did not account for the reduced stability observed in relation to schizotypal and autistic traits. These findings suggest that individuals with higher schizotypal and autistic traits struggle to form stable, lasting semantic predictions, which may contribute to difficulties in efficient language processing.

## Introduction

Schizophrenia spectrum disorders (SSD) and autism spectrum disorder (ASD) are diagnostically separate clinical conditions with distinct symptom criteria and clinical progressions^1,2^. While ASD is a neurodevelopmental disorder with an early onset which is usually diagnosed in childhood, SSD – including the diagnoses schizophrenia, other psychotic disorders and schizotypal personality disorder – typically develop later in adolescence or early adulthood. Additionally, ASD is often characterized by a stable or improving long-term prognosis whereas SSD are associated with persistent long-term impairments. Despite these differences, SSD and ASD show a considerable overlap in genetic predisposition, environmental risk factors and neurobiological alterations^1–4^. Importantly, both conditions also share symptom features, affecting various cognitive domains^3,5,6^. From a transdiagnostic perspective, it is therefore highly informative to study both cohorts together, especially in adults where these similarities are most noticeable. Among these shared clinical characteristics, language processing deficits, spanning impairments in processing grammatical, semantic, prosodic and pragmatic input^7–10^, are particularly relevant because of their central role in social interaction and daily functioning.

Generally, language processing is a highly dynamic process in which the brain must keep up with up to three words per second while simultaneously integrating the incoming information with the previous context. In recent decades, the hypothesis that the brain actively predicts upcoming input to efficiently process linguistic information has gained overwhelming support^11,12^. Behavioral studies show that predictable content is processed faster whereas unpredictable inputs are associated with costs such as increased reaction times^13–16^. This is supported by eye tracking studies demonstrating that the gaze is directed to predictable visual targets even before the auditory information is completely available^17–19^. Neurophysiological evidence from EEG studies shows that predictable sentences induce a larger negative semantic prediction potential^20–23^ and stronger alpha and beta desynchronization^22,24–26^, an effect which is gradually increasing for predictable compared to unpredictable contexts. Studies investigating the N400, i.e., a negative deflection of the EEG signal with a centro-parietal scalp distribution which peaks between 300 and 500 ms after word onset, reliably show that its amplitude is modulated by the expectancy of a critical word within its context^27^. Here, surprising or unexpected words elicit a more negative amplitude compared to predictable words which emphasizes the role of predictability during semantic language processing^28^. Critically, these electrophysiological responses which are associated with effective predictive language processing appear to be attenuated or even absent in individuals with ASD^29–37^ and individuals with SSD^38–43^.

**Fig. 1:**
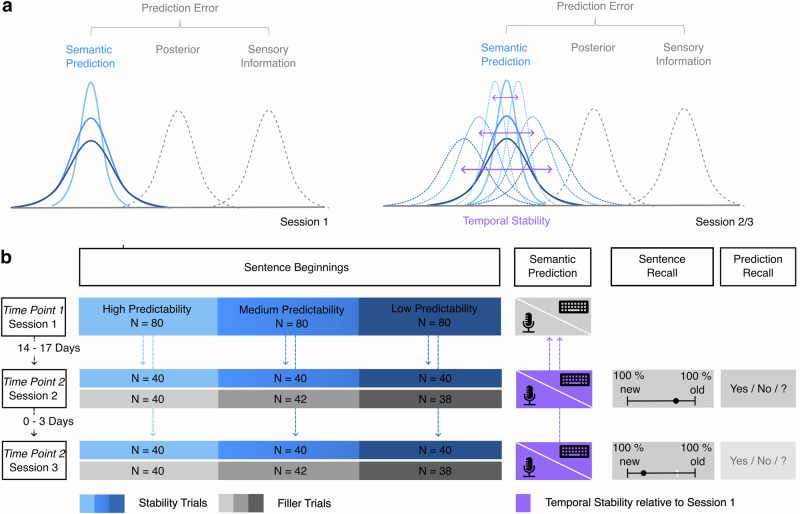
Task Design. **a** Schematic illustration of the task design within the predictive coding framework. Prior beliefs, in form of semantic predictions, are assessed in session 1, their temporal stability is either tested in session 2 and 3, as indicated by the dashed blue lines and violet arrows. It is hypothesized that the temporal stability (violet arrows) depends on the prior precision, and thus is higher for sentences that are predictable, and lower for sentences that are unpredictable. **b** Stability task. The experiment consisted of three sessions to measure the temporal stability of semantic predictions across two time points. In session 1 (time point 1), participants listened to 240 sentence beginnings of three levels of predictability in randomized order and were asked to predict the ending of the sentences. Semantic prior beliefs were recorded both as spoken and written responses. The sentences from session 1 (stability trials) were evenly split across the follow-up sessions 2 and 3 (time point 2), intermixed with new sentences (120 filler trials for each session), and were presented in fully randomized order. Participants were again asked to complete the sentences with their semantic prediction. After the recording, participants were asked to rate whether they had already heard the sentence in session 1 on a scale ranging from “100% not presented” to “100% presented”. If their confidence rating fell on the latter half of the scale (closer to “100% presented”), they were additionally asked whether they had provided the same response in in the first session as in the current session, with the response options “yes”, “no” or “I don’t remember” to assess prediction recall.

The central role of predictive processing in language aligns with the framework of hierarchical predictive coding. By capturing the hierarchical representation of language, ranging from phonetics to semantics and eventually pragmatics, it provides a computational account of how prior beliefs from higher levels (e.g., semantics) and their associated predictions guide language processing at lower level of sensory information (e.g., phonology)^11,12^. Predictive coding also offers a plausible framework for other cognitive domains such as perception, decision-making, memory or social cognition, suggesting a universal cognitive architecture in the brain^44^. While recent findings provide evidence for hierarchical predictions during language processing^45–48^, it remains unclear how (semantic) prior beliefs are initially formed^12^ and maintained or updated over time. So far, empirical findings indicate that prior beliefs are shaped by contextual predictability, with greater predictability leading to predictions with higher precision – a Bayesian measure reflecting the inverse variance of a probability distribution (Fig. 1a). For example, a highly predictable sentence beginning as “The swimmer jumped into the …” will elicit a very concrete semantic prediction of the sentence-final word “pool”, characterized by a narrow, highly precise probability distribution. In contrast, a less predictable sentence beginning like “The child hid the toy under the …” will allow for multiple plausible continuations, such as “bed”, “chair”, “table”, etc., resulting in a broader, less precise semantic prediction.

An open question, however, relates to whether and how these specific semantic prior beliefs change over time, i.e., do they remain temporarily stable or do they fluctuate from one time point to the next, and how this process is also influenced by their precision. Understanding the temporal stability of semantic prior beliefs is clinically relevant for two reasons: First, predictive coding approaches of SSD^49–54^ and ASD^55–57^ suggest that changes in Bayesian inference, specifically an abnormal weighting of prior beliefs and or sensory information may explain symptom development; and second, language processing alterations have been discusses as potential driving factors to the emergence of clinical symptoms in both disorders^58–62^.

Previous empirical research has linked symptoms of ASD, such as sensory overload, to an overweighting of low-level (bottom-up) sensory information^63,64^, resulting in uninformative sensory prediction error processing^65–68^. In SSD, overweighting of sensory information on lower levels^69,70^ and abnormal prediction error signaling^54,71–74^, which may explain the rigidity of prior beliefs, have been associated with the development of delusions as an explanation for these salient experiences. Additionally, SSD have also been characterized by an overweighting of high-level (top-down) prior beliefs^75–78^ which may override the sensory input from the environment, potentially accounting for the development of hallucinations.

While these studies indicate impairments in the integration of prior beliefs and sensory evidence in ASD and SSD, few have focused specifically on the formation and maintenance of prior beliefs over time. Therefore, investigating whether semantic prior beliefs are temporally stable or fluctuate across timepoints depending on their precision, could not only provide a deeper understanding of impairments in predictive language processing, but also add to predictive coding approaches of ASD and SSD.

To explore this, the present study examined whether the temporal stability of semantic predictions varies as a function of their precision and subclinical autistic and schizotypal trait levels in a healthy adult sample. In recent years, the disease conceptualization of both ASD and SSD has undergone a similar evolution, shifted from a categorical to a dimensional approach where subclinical personality traits in the general population exist along an etiological continuum that extends to severe clinical manifestations at the extreme ends^79,80^. Critically, alterations of both predictive coding mechanisms^77,78,81–89^ and predictive language processing alterations^38,39,90–93^ have also been identified using these subclinical trait measures, mirroring those observed in clinical populations with ASD and SSD. Therefore, focusing on an adult subclinical sample does not only help to capture the full spectrum of the disorders, but it also minimizes confounding factors such as medication or illness-duration.

In the present study, we used a preregistered online auditory stability task to assess whether participants, who were assessed on schizotypal and autistic traits, formed temporally stable predictions across two time points, following sentence beginnings of varying predictability. Predictability was mathematically defined as the Shannon entropy of the sentence beginning which quantifies the uncertainty about the remainder of the sentence (e.g., “The swimmer jumped into the …” represents low entropy/high predictability while “The park nearby has a large …” represents medium entropy/medium predictability and “The child hid the toy under the …” represents high entropy/low predictability^94^). Since semantic prior beliefs may be strongly shaped by mnemonic representations^95,96^, we explored whether the pattern of participants’ predictions might change depending on how well they remembered the previously presented sentences. Therefore, at the second time point we asked participants to recognize the previously presented sentences, which were intermixed with new sentences.

We hypothesized that temporal stability would depend on the predictability of the preceding sentence, with semantic predictions following highly predictable sentence beginnings being more stable over time compared to those for medium- or low-predictability sentence beginnings. In a recent study, we demonstrated that schizotypal traits are associated with significant overweighting of semantic prior beliefs during predictive language processing^85^. However, it is not straightforward to predict whether this overweighting also corresponds to greater stability of semantic predictions over time. On the one hand, the tendency to rely heavily on prior knowledge^77,85^, along with previous evidence of similar semantic processing alterations in SSD and ASD^37,42,43^ could imply more robust semantic predictions for increasing schizotypal and autistic traits. On the other hand, evidence from research on decision-making indicates that individuals with SSD^97–99^ and ASD^100,101^ are more random in their choices and update their prior beliefs in response to changing environments more rapidly^64,102–104^. This could point to an overall increase in the volatility of prior beliefs (i.e., unstable, frequently changing predictions) which may arise independently of the inference process itself and could induce less stable predictions. Taken together, while current research implies that both the use and the formation of predictions are suboptimal in subclinical traits of ASD and SSD, it remains unclear whether the temporal stability of semantic predictions is also affected.

## Methods

### Preregistration

The design of the present study was preregistered (https://osf.io/h4sq5). The final sample size was increased to allow for a more complex analysis using linear mixed models.

### Sample

We recruited 134 participants through advertisement at university departments, social media and word of mouth. Nineteen participants were excluded for the following reasons: drop-outs after the first session (*N* = 5), data loss in the first session due to technical difficulties (*N* = 1), a time gap between sessions 1 and 2, which exceeded the acceptable range of 14 to 17 days (*N* = 10), more than 10% missing responses per session (*N* = 1), and grammatical errors on over 10% of trials per session (*N* = 2). This resulted in a final sample size of 115 (90 female, 25 male) which included 92 complete data sets covering all three sessions, and 17 partial data sets including session 1 and 2, and 6 partial data sets including session 1 and 3. For the 23 partial data sets, six data sets from the second session and thirteen from the third session were lost due to technical issues. Additionally, four participants’ third session data sets were excluded due to a time gap of more than three days between sessions 2 and 3.

All participants indicated German as their native language with 18 participants stating an additional native language. Autistic and schizotypal personality traits were assessed before the start of the first session and once again after completing the third session of the experiment. Autistic traits were measured with the Autism Spectrum Quotient^105^ (AQ) and schizotypal traits with the Schizotypal Personality Questionnaire^106^ (SPQ; see Supplementary Methods for details). Table 1 shows an overview of the sample characteristics and subclinical measures. Subclinical measures were highly correlated between sessions and questionnaires (Supplementary Methods, Fig. S1). Prior to participation, participants were presented with detailed information outlining the study procedure, data protection, and their rights as participants and provided informed consent electronically via button press to initiate the online experiment. After completion, the participants received optional course credit. The study was approved by the Ethics Commission of the Technical University of Munich (approval numbers 786/20 S-SR).

**Table 1:** Summary statistics of demographic variables and subclinical questionnaire scores.

	*N*	Mean (SD)	Range
Age in years	115	21.97 (4.65)	18–59
AQ (Session 1)	115	15.67 (6.01)	5–32
AQ (Session 3)	98	16.62 (6.47)	6–35
SPQ (Session 1)	114	77.99 (39.31)	7–165
SPQ (Session 3)	97	77.63 (42.96)	5–205
Time gap between Session 1 and Session 2 in days	109	14.55 (0.95)	14–17
Time gap between Session 2 and Session 3 in days	98	1.35 (0.69)	0–3

Differences in sample size result from inclusion of partial data sets (see main text). The SPQ score is missing for one participant in session 1 and for one in session 3 due to technical difficulties.

*AQ* Autism Spectrum Quotient, *SPQ* Schizotypal Personality Questionnaire.

### Stability task

The stability task was implemented using jsPsych^107^ (v7.3.2;), and was hosted on Pavlovia (https://pavlovia.org). Participants could only perform the task on a desktop or laptop computer which was verified by a web browser check. To initiate the task, participants gave informed consent; and they completed an audio and microphone check. The task consisted of two time points. Time point 1 was comprised of one session, while timepoint 2 was comprised of two sessions. The task was explained at the beginning of each session using one practice trial with step-by-step instructions.

The task design is visualized in Fig. 1b. In each of the three sessions participants were asked to listen to 240 sentence beginnings of three levels of predictability (low, medium, and high) which were presented randomly in four blocks of 60 trials with self-timed breaks. During the auditory presentation of the sentence beginnings, a playback symbol served as a fixation icon in the center of the screen. Immediately afterwards, a microphone icon appeared as a cue for the participants to complete the sentence with the missing sentence-final word as quickly as possible. The recording of the participants’ prediction automatically started after a 50 ms delay and lasted for a fixed duration of 3500 ms. Participants were then asked to type the word they had uttered. At timepoint one (session 1), participants initiated the start of the next trial by clicking on “Next” or pressing the “Enter” key. At timepoint two (session 2 and 3), participants were additionally asked how confident they were that they had already heard the presented sentence in the first session on a scale ranging from “100% not presented” to “100% presented”, with an initial position at 0%. If the sentence recall rating deviated from the middle of the scale (0%) towards “100% presented”, participants were asked whether they had provided the same response in the current session as in the first with the response options “yes”, “no” or “I don’t remember” before the start of the next trial. As prediction recall performance is the primary focus of a different study, it was not used for the present analysis. Session 2 was made available 14 days after session 1 and had to be completed within 17 days after session 1. Session 3 was made accessible after the completion of the second session and had to be completed within 3 days after session 2.

### Stimuli

The stimuli used in this study were 480 audio recordings of spoken sentences. The sentences’ predictability was determined in an independent sample of participants. Here, a minimum of 40 and maximum of 80 individuals were asked to complete written sentences with one missing sentence-final word. Predictability was operationalized using Shannon entropy, which is a measure of the uncertainty about the outcome of a random variable. In sentence processing, entropy can be calculated at any word and expresses the uncertainty about the remainder of the sentence^108^. Entropy values were calculated with the 1.3.1 entropy package^109^ in R Studio^110^ and the maximum likelihood estimation method. Based on entropy categories used in a previous study^85^, 80 sentences of high predictability (entropy: 0–0.69), 80 sentences of medium predictability (entropy: 0.81–2.09) and 80 sentences of low predictability (entropy: 2.50–4) were selected from these 480 sentences as stability trials and were all presented in time point 1 (session 1) of the experiment (Fig. 1b). To assess the stability of semantic predictions across two time points, the 240 stability trials presented in time point 1 were each presented once more – either in session 2 or session 3, which both are part of time point 2. To enable this, the stability trials were split into two equal subsets of 120 trials. Each subset was then intermixed and counterbalanced with 120 of the remaining 240 sentence beginnings which served as filler trials. This splitting ensured that both session 2 and 3 of time point 2 contained 240 trials in total – 120 stability and 120 filler trials – allowing us to keep the number of sentence beginnings per session constant. The order of presentation of the two subsets was counterbalanced across participants so that each subset appeared equally often in session 2 or session 3. Within each session, the order of presentation of the stability and filler trials was fully randomized.

For the 240 filler trials, we aimed to present 80 sentences per predictability condition throughout the course of the experiment to match the proportion of the stability trials. However, an accidental imbalance resulted in 80 high predictability, 84 medium predictability, and 76 low predictability sentences (see Supplementary Methods and Table S1 for a comparison of stimulus characteristics).

### Statistical analysis

All statistical analyses were conducted in R Studio^110^ and visualized with the ggplot2 package^111^ (version 3.5.1). To define the temporal stability of semantic predictions, the semantic predictions of session 2 and 3 of the stability trials were compared to the semantic predictions from session 1. As audio recordings of the semantic predictions were occasionally missing or incomplete due to technical issues or the passing of the 3500 ms response window, we relied on the written responses for all participants to ensure consistency across the datasets and to avoid data loss. Discrepancy rates between spoken and written responses were low, ranging from 0% to 2.64% (mean = 0.24, SD = 0.38). Explorative correlation analyses revealed no significant correlation between deviation rates and schizotypal or autistic personality traits (Supplementary Methods, Fig. S2). Filler trials were not included in the analysis as they were only presented once throughout the experiment. Trials with stable predictions, i.e., if the semantic prediction reported in time point 2 was identical to the one reported in time point 1, were coded as 1, unstable predictions, i.e., if the semantic predictions reported in time point 2 was different to the one reported in time point 1, were coded as 0. To investigate the influence of sentence predictability, subclinical personality traits and memory performance on the temporal stability of semantic predictions, we performed generalized linear mixed models using the lme4 package^112^ with 5000 iterations (version 1.1 – 35.5) with the sentence’s predictability level (low, medium, high), the subclinical personality traits, the trial-level sentence recall performance (hit, miss) and their interactions as fixed effects. Subclinical personality traits were z-transformed for robustness and comparability, given the different maximum ranges between the SPQ (0–296) and AQ (0– 50) questionnaires. Participant and presented sentence beginning were included as random intercepts. Generalized linear mixed models were carried out separately for schizotypal and autistic traits.

$${\mathrm{stable}}_{\mathrm{SPQ}} \sim \mathrm{sentence}\,{{\mathrm{predictability}}_{\left(\mathrm{low},\,\mathrm{medium},\,\mathrm{high}\right)}}^{* }{\mathrm{SPQ}}^{* }\mathrm{sentence}\,{\mathrm{recall}}_{\left(\mathrm{hit},\,\mathrm{miss}\right)}+\left(1|\mathrm{subject}\right)+\left(1|\mathrm{sentence}\right)$$

$${\mathrm{stable}}_{\mathrm{AQ}} \sim {{\mathrm{sentence\; predictability}}_{\left(\mathrm{low},\mathrm{medium},\mathrm{high}\right)}}^{* }{\mathrm{AQ}}^{* }{\mathrm{sentence\; recall}}_{\left(\mathrm{hit},\mathrm{miss}\right)}+\left(1|\mathrm{subject}\right)+\left(1|\mathrm{sentence}\right)$$

Sentence recall performance (hit, miss, correct rejection, false alarm) was defined based on the accuracy of the participant’s subjective recall rating. A hit was defined as a confidence rating for stability trials that deviated from the midpoint toward “100% presented”, indicating that the participant correctly identified the sentence as old. A miss was defined as a rating for stability trials at or closer to “100% not presented”, indicating that the participant falsely judged the sentence as new. A false alarm was defined as a confidence rating for filler trials that deviated from the midpoint toward “100% presented”, indicating that the participant falsely judged the sentence as old. A correct rejection was defined as a rating for filler trials at or closer to “100% not presented”, indicating that the participant correctly identified the sentence as new.

As generalized linear mixed models were conducted on stability trials to investigate the temporal stability of semantic predictions, filler trials were not included in the analyses. Thus, only hits and misses were considered in the reported models.

Tukey adjusted post hoc tests and simple slope analyses were conducted to follow up main effects and interaction effects using the emmeans package^113^ (version 1.10.4) and the interactions package^114^ (version 1.2.0).

## Results

We performed two generalized linear mixed models with sentence predictability (low, medium, high), subclinical personality traits (AQ/SPQ), sentence recall performance (hit, miss), and their interactions as fixed effects and the participant and sentence as random intercepts, to investigate the temporal stability of semantic predictions.

**Fig. 2:**
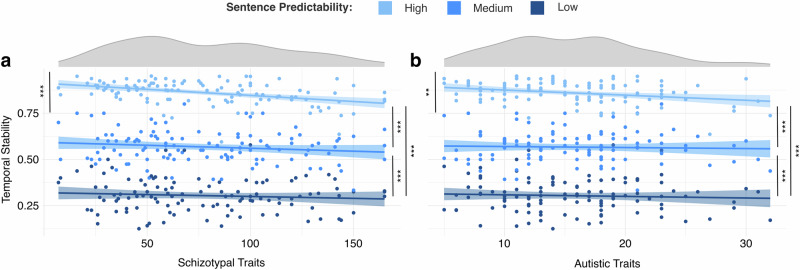
Generalized linear mixed model results visualized as scatter plots. Scatter plots **a**, **b** show graded effect of the sentence beginnings’ predictability level on the temporal stability of semantic predictions, with higher predictability associated with more stable semantic predictions. **a** Higher schizotypal and **b** higher autistic traits are linked to decreased temporal stability in highly predictable contexts; **p <0.01; ***p < 0.001.

As shown in Table 2, there was a significant main effect of sentence predictability in all predictability levels. Paired Tukey corrected post hoc comparisons showed that there was a graded effect of sentence predictability with higher stability values in the high predictability condition than in the medium predictability condition (SPQ: 1.85, 95% CI = [1.61; 2.09]; AQ: 1.84, 95% CI = [1.60; 2.07]), and higher stability values in the medium predictability condition than in the low predictability condition (SPQ: 1.19, 95% CI = [0.96; 1.42]; AQ: 1.18, 95% CI = [0.95; 1.41]) (Fig. 2a, b).

**Table 2:** Results of GLMM on temporal stability including memory performance.

$$\mathrm{stable} \sim {\mathrm{predictability}}^{* }\mathrm{AQ}/{\mathrm{SPQ}}^{* }\mathrm{sentence\; recall}+(1|\mathrm{sentence})+(1|\mathrm{participant})$$								
	SPQ				AQ			
Fixed Effects	Beta [95% CI]	SE	Z value	*p*	Beta [95% CI]	SE	Z value	*p*
	2.23 [2.07; 2.39]	0.08	26.92	**<0.001**	2.22 [2.06; 2.38]	0.08	26.82	**<0.001**
Medium Predictability	−1.65 [−1.85; −1.44]	0.10	−15.82	**<0.001**	−1.63 [−1.83; −1.42]	0.10	−15.75	**<0.001**
Low Predictability	−2.66 [−2.86; −2.45]	0.11	−25.30	**<0.001**	−2.65 [−2.85; −2.43]	0.10	−25.24	**<0.001**
SPQ / AQ	−0.21 [−0.31; −0.12]	0.05	−4.32	**<0.001**	−0.14 [−0.23; −0.04]	0.05	−2.88	**0.004**
Sentence Recall	−0.62 [−0.77; −0.47]	0.08	−8.00	**<0.001**	−0.63 [−0.78; −0.48]	0.08	−8.35	**<0.001**
Medium Predictability x SPQ / AQ	0.18 [0.08; 0.27]	0.05	3.64	**<0.001**	0.16 [0.06; 0.25]	0.05	3.31	**<0.001**
Low Predictability x SPQ / AQ	0.18 [0.08; 0.27]	0.05	3.50	**<0.001**	0.08 [−0.02; 0.18]	0.05	1.62	0.105
Medium Predictability x Sentence Recall	−0.43 [−0.61; −0.25]	0.09	−4.60	**<0.001**	−0.42 [−0.60; −0.24]	0.09	−4.55	**<0.001**
Low Predictability x Sentence Recall	−0.79 [−0.98; −0.60]	0.10	−8.09	**<0.001**	−0.76 [−0.95; −0.57]	0.10	−7.92	**<0.001**
SPQ/AQ x Sentence Recall	−0.06 [−0.21; 0.09]	0.08	−0.77	0.441	0.00 [−0.14; 0.15]	0.07	0.04	0.964
Medium Predictability x SPQ / AQ x Sentence Recall	0.03 [−0.15; 0.21]	0.09	0.32	0.752	−0.10 [−0.28; 0.07]	0.09	−1.13	0.258
Low Predictability x SPQ / AQ x Sentence Recall	0.11 [−0.08; 0.30]	0.09	1.16	0.247	0.09 [−0.10; 0.27]	0.09	0.93	0.350
**Random Effects**	**Variance**		**SD**		**Variance**		**SD**	
Sentence	0.32		0.57		0.32		0.57	
Participant	0.09		0.31		0.10		0.31	

The generalized linear mixed models also revealed a significant effect of sentence recall performance in the high predictability baseline condition and a significant interaction effect between the sentence recall performance and the medium predictability and low predictability condition. Sentence recall performance assessed the participants’ accuracy to recognize whether a sentence had been presented at the first time point (for further details see Methods – Statistical Analysis). Simple slope analyses revealed that temporal stability was significantly lower during miss trials compared to hit trails across conditions and that this effect was even stronger in the medium predictability (SPQ: −1.05, 95% CI = [−1.15; −0.94], SE = 0.06, z = −18.75, *p* < 0.001; AQ: −1.06, 95% CI = [−1.17; −0.95], SE = 0.06, z = −19.06, *p* < 0.001) and low predictability condition (SPQ: −1.39, 95% CI = [−1.52; −1.28], SE = 0.06, z = −22.64, *p* < 0.001; AQ: −1.37, 95% CI = [−1.49; −1.25], SE = 0.06, z = −23.51, *p* < 0.001) compared to the high predictability condition (SPQ: −0.62, 95% CI = [−0.77; −0.47], SE = 0.08, z = −8.11, *p* < 0.001; AQ: −0.63, 95% CI = [−0.78; −0.48], SE = 0.08, z = −8.43, *p* < 0.001).

In both models, we also observed a significant effect of the subclinical personality traits in the high predictability condition as well as a significant interaction effect between the subclinical personality traits and the medium and low predictability conditions. Follow-up simple slope analyses showed there was a significant decrease of the temporal stability of semantic predictions in the high predictability condition for increasing SPQ (Fig. 2a) and AQ values (Fig. 2b; SPQ: −0.21, 95% CI = [−0.31;−0.12], SE = 0.05, z = −4.33, *p* < 0.001; AQ: −0.14, 95% CI = [−0.24; −0.05], SE = 0.05, z = −2.88, *p* = 0.004), whereas there was no significant change of temporal stability in the medium (SPQ: −0.04, 95% CI = [−0.12; 0.04], SE = 0.04, z = −0.89, *p* = 0.373; AQ: 0.02, 95% CI = [−0.06; 0.10], SE = 0.04, z = 0.44, *p* = 0.657) and low predictability conditions (SPQ: −0.04, 95% CI = [−0.12, 0.05], SE = 0.04, z = −0.88, *p* = 0.380; AQ: −0.06, 95% CI = [−0.14; 0.02], SE = 0.04, z = −1.39, *p* = 0.163).

Critically, the generalized linear mixed models neither revealed a significant interaction effect between the subclinical personality traits and the sentence recall performance, nor a triple interaction between the sentence predictability levels, which indicates that observed decrease in the temporal stability in the high predictability condition for increasing schizotypal and autistic traits cannot be explained by deficits in sentence recall performance.

To control for outliers in age and for additional native languages reported alongside German, exploratory control analyses were conducted. Excluding outliers in age, based on the median absolute deviation rule, did not change the results obtained from the main analysis. Excluding participants, who reported additional native languages other than German, produced the same overall pattern of results as in the main analysis for schizotypal traits. For autistic traits, the effect of decreasing stability in the highly predictable condition disappeared, likely because participants with additional native languages had significantly higher SPQ and AQ scores (SPQ: *W* = 519.5, *p* = 0.008; AQ: *W* = 441.5, *p* < 0.001) (see Supplementary Results, Tables S2 and S3). As sentence recall performance was assessed through the accuracy of subjective sentence recall ratings we performed additional explorative correlation analyses between recall ratings, memory measures and schizotypal and autistic personality traits. There was no significant correlation between sentence recall ratings and subclinical personality traits for the different memory measures (hits, misses, correct rejections, false alarms; see Supplementary Results, Fig. S3). In addition, there was no correlation between hit rates and false alarm rates and the subclinical personality traits (see Supplementary Results, Fig. S4).

## Discussion

In this study, we investigated the temporal stability of semantic predictions during language processing in individuals with subclinical schizotypal and autistic traits using an online auditory stability task. Our findings show that temporal stability was gradually influenced by the predictability of the preceding context, with higher predictability leading to greater stability. Notably, increasing subclinical schizotypal and autistic traits were associated with reduced temporal stability of semantic predictions in highly predictable contexts. Furthermore, mnemonic processes played a significant role in shaping stability, with poorer sentence recall being associated with greater instability, particularly in the medium and low predictability contexts. Memory performance, however, did not explain the reduced temporal stability with increasing subclinical personality traits.

Recent research has highlighted the critical role of semantic predictions in guiding processing as language unfolds^11,12^. Within the framework of predictive coding, the given context is expected to form a high-level semantic prediction with its contextual predictability shaping its precision or concreteness. While previous empirical studies have primarily focused on how semantic predictions are integrated with incoming sensory evidence, it is unknown how stable the predictions are as time progresses and whether their stability is also shaped by the predictability of the preceding sentence. By employing sentence beginnings of three different predictability levels, we demonstrated that the temporal stability of semantic predictions is strongly influenced by the predictability of the preceding context. In Bayesian terminology, this implies that more precise semantic prior beliefs following highly predictable sentences remain more robust over time.

In a recent study^85^, we found that subclinical schizotypal traits were associated with an overweighting of semantic prior beliefs compared to sensory evidence, supporting previous studies, identifying similar imbalances in the weighting of prior beliefs or sensory evidence in subclinical populations^77,78,81–84,86–89^ as in clinical cohorts^63,64,69,70,75–78^. Based on this, one may have argued that this overreliance would manifest in a higher temporal stability of semantic prior beliefs with increasing schizotypal and autistic traits. Instead, we observed that increasing schizotypal and autistic traits were associated with reduced temporal stability of semantic predictions, particularly following highly predictable contexts. Our paradigm, measuring the stability of semantic prior beliefs, does not allow for any conclusions on altered weighting of prior beliefs and sensory evidence over time. However, our findings may point towards an important temporal differentiation of predictive coding mechanisms: While our earlier study indicates that semantic predictions are overweighted during moment-to-moment inference and integration with incoming sensory evidence, the present result may reflect an additional broader instability in maintaining semantic predictions over time. This suggests that even if semantic predictions are overweighted locally, they may still lack robustness across longer timescales. In line with this, recent research focusing on how individuals with SSD and ASD adapt to changing environments suggests that individuals on both spectra tend to perceive their environment as overly volatile, leading to exaggerated belief updating^64,102–104^. Although our task did not manipulate volatility or rigidity of prior beliefs and, therefore, cannot directly probe belief updating in response to changing feedback from the environment, our finding that increasing subclinical schizotypal and autistic traits are associated with less robust semantic predictions may indirectly reflect these alterations observed in clinical cohorts. Taken together, our findings highlight the relevance to consider not only how priors are weighted in relation to incoming sensory information but also how they develop and persist over time, particularly in contexts where stable predictions typically guide cognitive processes most efficiently. This could be a crucial factor in understanding fluctuations in cognitive and perceptual symptoms, particularly in SSD where symptom expression often varies over time.

Our observation that temporal instability of semantic predictions specifically emerges in predictable rather than unpredictable contexts, as a function of increasing subclinical traits, is consistent with electrophysiological findings. These studies report deficits in processing predictable or semantically related input in individuals with SSD and ASD, rather than alterations in processing unpredictable or semantically unrelated inputs^39,115–117^. In line with this, we found in a recent analysis which was based on a sentence paradigm involving the same predictability levels as in the present study that high and medium predictability sentences elicit more negative N400 amplitudes with increasing subclinical schizotypal and autistic traits (Sterner et al., in preparation). Related to this, studies using verbal fluency tasks report reliably that individuals with SSD^118^ show impairments in forming semantic associations. Some studies also identify these alterations in individuals with ASD; however, the empirical evidence is inconsistent^119^. It is therefore also plausible that the difficulties in maintaining predictions over time could stem from an impairment in forming meaningful and efficient predictions at a semantic level. This may lead individuals with higher autistic and schizotypal traits to sample their predictions from semantic space more frequently which might not be evident in the medium predictable and unpredictable condition, where variability is higher even among individuals with low trait levels.

Given the high correlation between schizotypal and autistic traits in our sample, one may argue that using subclinical personality traits as markers makes it difficult to disentangle alterations specific to ASD and SSD and translate findings to clinical cohorts. However, previous research suggests that such overlap is not merely a limitation of subclinical traits^120^, but rather a feature inherent to the clinical stages of both disorders, particularly regarding shared negative and cognitive symptom dimensions. Moreover, similar semantic processing alterations have been observed in both SSD and ASD^37,42,43^, and also in individuals with schizotypal^38,39,92,93^ and autistic traits^90,91^, supporting the notion of common underlying mechanisms between disorders but also along the subclinical-clinical dimensions. Our results may therefore reflect another shared alteration in language processing rather than an artifact of subclinical trait correlation. As our sample was recruited from the general population, covering a broad distribution of subclinical traits, future research should focus on high-scoring individuals particularly and investigating risk populations and clinical cohorts to disentangle disorder-specific mechanisms and to clarify the generalizability of our findings to individuals on the clinical ends of these spectra.

While our findings highlight shared alterations in temporal stability of semantic predictions in autistic and schizotypal traits, they also raise questions about influencing factors. Age, for instance did not significantly impact the results when outliers were excluded. However, given evidence that predictive processing in language may change over the lifespan^121,122^, future research using broader age samples and longitudinal designs could provide insights in how these mechanisms shift during aging.

Similarly, while controlling for additional native languages other than German eliminated the effect of decreasing stability in the highly predictable condition for autistic traits, follow-up analyses demonstrated that both autistic and schizotypal traits were significantly higher in the subsample with multiple native languages. This suggests that the effect of schizotypal traits is more robust, whereas the relationship between autistic traits and semantic prediction stability may be more sensitive to the individual linguistic background. Although having multiple native languages, in combination with higher autistic traits could contribute to additional impairments in temporal stability of semantic predictions, growing evidence suggests that bilingualism does not negatively impact language processing in ASD^123^. Future studies with larger and more balanced samples of mono- and multilingual speakers could help clarify these effects.

Given that memory impairments are one of the most robust cognitive symptom features in SSD^124,125^ and ASD^126^, one obvious explanation for the observed decrease in temporal stability could have been a failure to form accurate memory representations of the presented sentences which could also be present with increasing subclinical traits. To investigate this, we included a matching number of sentences which the participants have not encountered before in the second time point. For these filler trials, we aimed to present an equal number of 80 sentences per predictability condition (high, medium, low), though the final distribution was slightly imbalanced with 80 high predictability, 84 medium predictability, and 76 low predictability sentences. The linear mixed models did not reveal an interaction effect between personality traits, sentence recall performance and sentence predictability on the temporal stability of semantic predictions. This suggests that the decreased temporal stability observed in the predictable condition cannot simply be attributed to poorer recall of the presented sentences by individuals with higher schizotypal and autistic traits. As sentence recall performance was defined based on the accuracy of subjective sentence recall ratings and studies have found evidence for metamemory biases in both schizotypy and SSD^127,128^, with less pronounced biases observed in ASD^129^, we carried out explorative correlation analyses between memory recall ratings and subclinical personality traits for hits, misses, false alarms and correct rejections. Additionally, we inspected correlations between hit and false alarm rates and subclinical personality traits. However, we did not observe any changes in recall ratings or memory performance measures with increasing subclinical personality traits. These results support our interpretation that the reduced temporal stability is a result of impaired predictive language processing rather than memory impairments or metacognitive biases.

Across the whole sample, however, we observed that the mnemonic representation of the presented sentence beginnings influenced the robustness of semantic predictions. This is to be expected given that language and memory, despite being conceptualized as distinct cognitive domains, are heavily intertwined not only functionally but also neurobiologically^95,96^. Specifically, we found that temporal stability decreased when participants failed to recognize the presented sentence from the first time point. This cost in temporal stability was especially pronounced for sentence beginnings with medium and low predictability. Prior studies investigating the interaction between memory performance and predictability have shown that predictable sentence-final words^121,130,131^ and sentence-final words which strongly violate predictions^132–134^ are recalled more effectively, indicating that predictability, and also its violation, modulates how well verbal information is encoded and retrieved. This effect has also been shown for other modalities using a wide range of paradigms and stimuli^135–138^. Our findings extend this body of research by demonstrating that the interplay between predictability and memory formation not only influences recall but also affects the temporal stability of semantic predictions.

### Limitations

Conducting experiments with auditory stimuli in an online as opposed to a laboratory setting may enhance ecological validity, particularly when studying language processing in everyday environments. To minimize potential limitations inherent to these designs, we implemented several preventive measures: Participants were explicitly instructed to wear headphones and complete the experiment in a quite environment. Before initiating the experiment, participants completed technical checks to verify headphone and microphone functionality. Volume checks were performed which allowed participants to adjust the sound volume, reducing the risk of poor audibility affecting comprehension. Despite these precautions, non-compliance or attentional lapses during the experiment cannot be entirely ruled out. Therefore, we applied strict exclusion criteria according to which a participant would be excluded for producing grammatical errors or missing more than 10% of the trials per session. Based on these criteria, only three participants had to be excluded, indicating that 115 out of 118 participants were compliant and engaged throughout the task. The sample was predominantly female and homogenous in educational background which may limit the generalizability of our findings but simultaneously allows for more direct conclusions within this controlled sample as especially literacy has been shown to affect predictive strategies during language processing^139^. Finally, it should be acknowledged that predictions and mnemonic representations are continuously shaped by individual experiences, broader contextual information, and world knowledge, all of which can hardly be controlled for in an experimental setting.

## Conclusion

In the present study, we investigated the temporal stability of semantic prior beliefs in an auditory stability paradigm. We found that the temporal stability of predictions gradually increased with increasing predictability of the preceding context. Our results show that increasing subclinical schizotypal and autistic traits were associated with decreasing robustness of semantic predictions following highly predictable sentences which could not be explained by altered sentence recall performance. Taken together, this points to a broader underlying deficit to form robust predictions to guide language processing, which needs to be validated in clinical samples.

## Supplementary information

Supplemental Material

## Data Availability

Data and code are available under https://osf.io/8jaf4/.

## Code availability

Data and code are available under https://osf.io/8jaf4/.
