# Supplementary material for: Temporal stability of semantic predictions in subclinical autistic and schizotypal personality traits
Source: Schizophrenia (Heidelb). 2025 Jul 19;11(1):103. doi: 10.1038/s41537-025-00643-9 (PMC12276268; doi:10.1038/s41537-025-00643-9)
Supplement: Supplementary file 1 — Supplemental Material [file 41537_2025_643_MOESM1_ESM.docx]

**Supplementary Material**

**Supplementary Methods**

**Subclinical Questionnaires**

**Autistic Spectrum Quotient.** The Autism Spectrum Quotient (AQ) is a 50-item self-report questionnaire designed to assess five different facets of autistic spectrum traits (social skills, attention, attention to detail, communication, imagination.^1^ The AQ is administered using a 4-point Likert scale format (strongly agree, somewhat agree, somewhat disagree, strongly disagree). A binary scoring system is used, with the presence of autistic traits, either mild or severe, scoring one point and the opposite scoring zero, resulting in a maximum score of 50. The items are counterbalanced so that half of the items are worded to produce a disagreement response and the other half to produce an agreement response in a high-scoring individual. In the present study, the AQ was administered in a German translated version before the start of the first session and again after completing the third session of the experiment.

**Schizotypal Personality Questionnaire.** The Schizotypal Personality Questionnaire (SPQ) is a 74-item self-report questionnaire designed to capture schizotypal traits in accordance with the Diagnostic and Statistical Manual of Mental Disorders, 3rd Edition, Revised (DSM-III-R) criteria for schizotypal personality disorder symptoms.^2^ The questionnaire measures nine dimensions of schizotypy, which can be assigned to a three-factor structure.^3^ This structure comprises positive traits (ideas of reference, magical thinking, unusual perceptual experiences, paranoia), negative traits (lack of close friends, constricted affect, social anxiety) and disorganized traits (odd speech, odd behavior). As a modified 5-point Likert scale version of the SPQ was demonstrated to enhance the sensitivity of detecting schizotypy traits in comparison to a dichotomous version (Wuthrich & Bates, 2005), the present study used an authorized German translation of the SPQ^4^ with the response criteria strongly disagree (0), disagree (1), neutral (2), agree (3), strongly agree (4). Based on this, an overall sum score and sum scores for the three factors were calculated. Nine dimensions can be calculated by summing the individual responses for the respective items.

**Heatmap Subclinical Questionnaires**

**
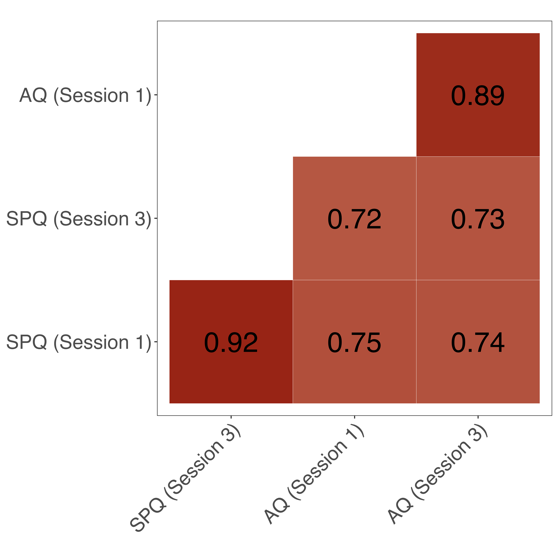
**

***Figure S1 Heatmap of Subclinical Questionnaire Scores.*** *Schizotypal and autistic traits were assessed before the start of session 1 and once after the completion of session 3. Pearson’s correlation analyses show that the scores are highly correlated between sessions and scores.*

**Stimuli**

| **Table S1:** Comparison of characteristics of repeated and unrepeated sentences | | | | | | |
| --- | --- | --- | --- | --- | --- | --- |
|  | Entropy (nats) | | | Sentence length (words) | | |
|  | Stability  Trials | Filler  Trials | Statistical  Comparison | Stability  Trials | Filler  Trials | Statistical  Comparison |
|  | Mean (SD) | Mean (SD) |  | Mean (SD) | Mean (SD) |  |
| Low | 0.27 (0.22) | 0.34 (0.24) | t(155.45) = -1.79,  p = .076 | 6.69  (1.58) | 6.85  (1.77) | t(154.76) = -0.60,  p = .548 |
| Medium | 1.49 (0.37) | 1.47 (0.29) | t(155.06) = -0.16,  p = .871 | 7.06  (1.27) | 6.74  (1.77) | t(151.7) =  -1.27,  p = .206 |
| High | 3.06 (0.38) | 2.78 (0.42) | **t(152.31) = 3.91,**  **p < .001** | 7.64  (1.54) | 7.13  (1.54) | **t(151.7) = -2.09,**  **p = .038** |

**Discrepancy rates between spoken and written responses and subclinical personality traits**

***
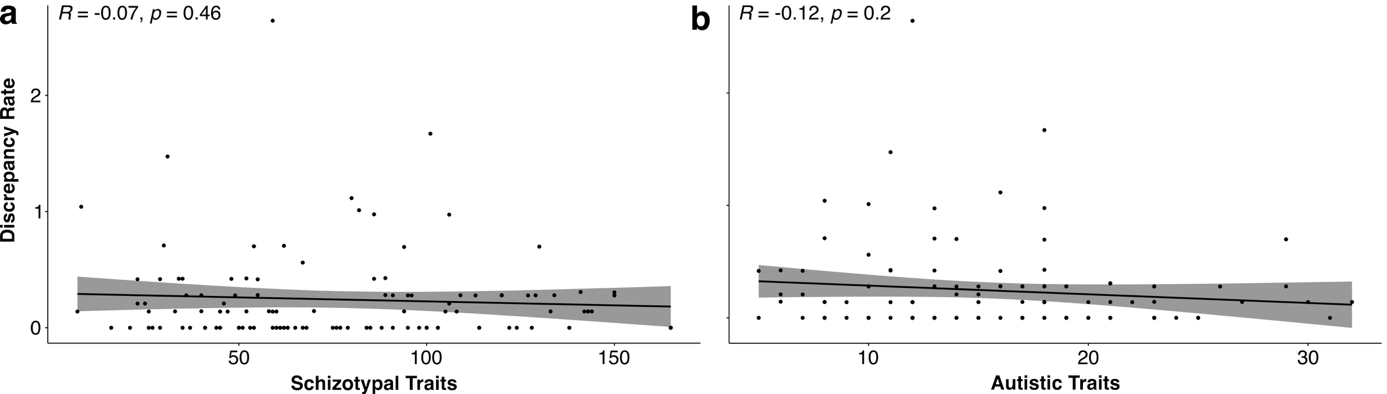
***

***Figure S2 Correlation Analyses between Discrepancy Rates and Schizotypal and Autistic Traits.*** Discrepancy rates of spoken and written semantic predictions were calculated as the percentage of the differing responses relative to the total number of trials per participant. Pearson correlations between discrepancy rates and (a) schizotypal traits and (b) autistics traits did not reveal significant correlations.

**Supplementary Results**

**Reanalysis of the GLMM without participants with second native language**

To investigate the effect of a second native language on the temporal stability of semantic predictions, we reperformed the two generalized linear mixed models with sentence predictability (low, medium, high), subclinical personality traits (AQ/SPQ), recall performance (hit, miss), and their interactions as fixed effects and the participant and sentence as random intercepts after excluding participants with additional native languages other than German (N = 18). Except for the interaction effect between AQ and the high predictability baseline condition, the overall pattern of results remained the same as in the main analysis.

As shown in **Table S2**, there was a significant main effect of sentence predictability in all predictability levels. Paired Tukey corrected post hoc comparisons showed that there was a graded effect of sentence predictability with higher stability values in the high predictability condition than in the medium predictability condition (SPQ: 1.87, 95% CI = [1.62; 2.11]; AQ: 1.85, 95% CI = [1.61; 2.10]), and higher stability values in the medium predictability condition than in the low predictability condition (SPQ: 1.16, 95% CI = [0.93; 1.40]; AQ: 1.16, 95% CI = [0.92; 1.39]).

The generalized linear mixed models also revealed a significant effect of recall performance in the high predictability baseline condition and a significant interaction effect between the recall performance and the medium predictability and low predictability condition. Simple slope analyses revealed that temporal stability was significantly lower during miss trials compared to hit trails across conditions and that this effect was even stronger in the medium predictability (SPQ: -1.04, 95% CI = [-1.16; -0.92], SE = 0.06, z = -17.01, p < .001; AQ: -1.05, 95% CI = [-1.17; -0.93], SE = 0.06, z = -17.32, p < .001) and low predictability condition (SPQ: -1.38, 95% CI = [-1.52; -1.25], SE = 0.07, z = -20.79, p < .001; AQ: -1.37, 95% CI = [-1.50; -1.24], SE = 0.07, z = -20.75, p < .001) compared to the high predictability condition (SPQ: -0.67, 95% CI = [-0.84; -0.51], SE = 0.08, z = -7.93, p < .001; AQ: -0.69, 95% CI = [-0.85; -0.53], SE = 0.08, z = -8.39, p < .001).

For SPQ, we also observed a significant effect of the subclinical personality traits in the high predictability condition as well as a significant interaction effect between the subclinical personality traits and the medium and low predictability conditions. Follow-up simple slope analyses showed there was a significant decrease of the temporal stability of semantic predictions in the high predictability condition for increasing SPQ (-0.19, 95% CI = [-0.31; -0.07], SE = 0.06, z = -3.09, p = .002), whereas there was no significant change of temporal stability in the medium (-0.06, 95% CI = [-0.15; 0.04], SE = 0.05, z = -1.19, p = .233) and low predictability conditions (SPQ: -0.04, 95% CI = [-0.14; 0.06], SE = 0.05, z = -0.85, p = .394). For AQ, there was only a significant interaction effect in the medium predictability condition. However, follow-up simple slope analyses showed there was no significant change of the temporal stability of semantic predictions in any condition for increasing AQ (high predictability: -0.92, 95% CI = [-0.21; 0.03], SE = 0.06, z = -1.49, p = .136; medium predictability: 0.04, 95% CI = [-0.06; 0.13], SE = 0.05, z = 0.74, p = 0.460, low predictability: -0.02, 95% CI = [-0.12; 0.08], SE = 0.05, z = -0.44, p = .661).

The generalized linear mixed models neither revealed a significant interaction effect between the subclinical personality traits and the recall performance, nor a triple interaction between the sentence predictability levels, which indicates that observed decrease in the temporal stability in the high predictability condition for increasing schizotypal traits cannot be explained by deficits in recall performance.

To investigate the changes of results compared to the main analysis, we conducted Wilcoxon t-tests to compare the mean AQ and SPQ values between participants with only German as native language and those who also reported additional ones. We used Wilcoxon t-tests as the normality assumption for SPQ in the group of participants with only German as a native language was violated (*W* = 0.97, *p* = 0.024) as well as the assumption of homogeneity of variances for SPQ (*F*(1, 112) = 4.85, *p* = 0.030). The results revealed that both SPQ and AQ values were significantly higher in participants with additional native languages (SPQ: mean = 105.56, SD = 51.50; AQ: mean = 20.72, SD = 7.06) compared to those with German only (SPQ: mean = 72.82, SD = 34.54; AQ: mean = 14.73, SD = 5.33), SPQ: *W* = 519.5, *p* = 0.008; AQ: *W* = 441.5, *p < .001.*

| **Table S2: Results of GLMM on temporal stability including memory performance** | | | | | | | | |
| --- | --- | --- | --- | --- | --- | --- | --- | --- |
| stable ~ predictability * AQ/SPQ * recall performance + (1\|sentence) + (1\|participant) | | | | | | | | |
|  | **SPQ** | | | | **AQ** | | | |
| **Fixed Effects** | **Beta**  **[95% CI]** | **SE** | **Z value** | **p** | **Beta**  **[95% CI]** | **SE** | **Z value** | **p** |
|  | 2.28  [2.07; 2.39] | 0.08 | 26.55 | **< .001** | 2.28  [2.11; 2.45] | 0.09 | 26.49 | **< .001** |
| Medium Predictability | -1.68  [-1.85; -1.44] | 0.11 | -15.62 | **< .001** | -1.66  [-1.87; -1.45] | 0.11 | -15.50 | **< .001** |
| Low Predictability | -2.66  [-2.86; -2.45] | 0.11 | -24.56 | **< .001** | -2.66  [-2.88; -2.45] | 0.11 | -24.56 | **< .001** |
| SPQ / AQ | -0.19  [-0.31; -0.12] | 0.06 | -3.09 | **.002** | -0.09  [-0.21; 0.03] | 0.06 | -1.49 | **.136** |
| Recall Performance | -0.68  [-0.77; -0.47] | 0.08 | -8.07 | **< .001** | -0.69  [-0.85; -0.53] | 0.08 | -8.44 | **< .001** |
| Medium Predictability * SPQ / AQ | 0.13  [0.08; 0.27] | 0.06 | 2.10 | **.035** | 0.13  [0.01;  0.25] | 0.06 | 2.04 | **.041** |
| Low Predictability * SPQ / AQ | 0.15  [0.08; 0.27] | 0.06 | 2.29 | **.022** | 0.07  [-0.06; 0.19] | 0.06 | 1.09 | .276 |
| Medium Predictability * Recall Performance | -0.36  [-0.61; -0.25] | 0.10 | -3.52 | **< .001** | -0.36  [-0.56; -0.16] | 0.10 | -3.61 | **< .001** |
| Low Predictability * Recall Performance | -0.70  [-0.98; -0.60] | 0.11 | -6.65 | **< .001** | -0.66  [-0.87; -0.46] | 0.10 | -6.40 | **< .001** |
| SPQ/AQ * Recall Performance | -0.06  [-0.21; 0.09] | 0.09 | -0.71 | .475 | -0.01  [-0.18; 0.16] | 0.09 | -0.12 | .903 |
| Medium Predictability * SPQ / AQ * Recall Performance | 0.11  [-0.15; 0.21] | 0.11 | 0.98 | .326 | -0.08  [-0.30; 0.13] | 0.11 | -0.77 | .440 |
| Low Predictability * SPQ / AQ * Recall Performance | 0.17  [-0.08; 0.30] | 0.13 | 1.49 | .137 | 0.14  [-0.08; 0.36] | 0.11 | 1.29 | .198 |
| **Random Effects** | **Variance** | | **SD** | | **Variance** | | **SD** | |
| Sentence | 0.32 | | 0.57 | | 0.32 | | 0.57 | |
| Participant | 0.08 | | 0.28 | | 0.08 | | 0.29 | |

**Reanalysis of the GLMM after outlier exclusion age**

As descriptive analyses revealed a large range in the participants’ age, we conducted an outlier analysis using the median absolute deviation rule with a threshold of 2.5. Based on this, five individuals aged 29, 32 (N = 2), 36 and 59 were excluded, and the main analysis was conducted without the identified outliers. We again performed two generalized linear mixed models with sentence predictability (low, medium, high), subclinical personality traits (AQ/SPQ), recall performance (hit, miss), and their interactions as fixed effects and the participant and sentence as random intercepts to investigate the temporal stability of semantic predictions. As shown in **Table S3**, excluding outliers in age did not change the results obtained from the main analysis.

|  | **Table S3: Results of GLMM on temporal stability including memory performance** | | | | | | | | | |
| --- | --- | --- | --- | --- | --- | --- | --- | --- | --- | --- |
|  | stable ~ predictability * AQ/SPQ * recall performance + (1\|sentence) + (1\|participant) | | | | | | | | | |
|  | | **SPQ** | | | |  | **AQ** | | | |
| **Fixed Effects** | | **Beta**  **[95% CI]** | **SE** | **Z value** | **p** | **Beta**  **[95% CI]** |  | **SE** | **Z value** | **p** |
|  | | 2.24  [2.08; 2.40] | 0.08 | 26.96 | **< .001** | 2.23  [2.06; 2.39] |  | 0.08 | 26.84 | **< .001** |
| Medium Predictability | | -1.65  [-1.86; -1.45] | 0.10 | -15.97 | **< .001** | -1.64  [-1.84; -1.44] |  | 0.10 | -15.88 | **< .001** |
| Low Predictability | | -2.66  [-2.86; -2.45] | 0.10 | -25.40 | **< .001** | -2.65  [-2.85; -2.44] |  | 0.10 | -25.34 | **< .001** |
| SPQ / AQ | | -0.20  [-0.03; -0.11] | 0.05 | -4.06 | **< .001** | -0.12  [-0.22; -0.02] |  | 0.05 | -2.42 | **.015** |
| Recall Performance | | -0.66  [-0.81; -0.51] | 0.08 | -8.41 | **< .001** | -0.67  [-0.82; -0.52] |  | 0.08 | -8.74 | **< .001** |
| Medium Predictability * SPQ / AQ | | 0.16  [0.07; 0.26] | 0.05 | 3.32 | **< .001** | 0.14  [0.04; 0.23] |  | 0.05 | 2.80 | **.005** |
| Low Predictability * SPQ / AQ | | 0.17  [0.07; 0.27] | 0.05 | 3.26 | **.001** | 0.06  [-0.04; 0.17] |  | 0.05 | 1.26 | .207 |
| Medium Predictability * Recall Performance | | -0.37  [-0.56; -0.18] | 0.09 | -3.91 | **< .001** | -0.37  [-0.55; -0.18] |  | 0.09 | -3.91 | **< .001** |
| Low Predictability * Recall Performance | | -0.73  [-0.92; -0.53] | 0.10 | -7.37 | **< .001** | -0.70  [-0.90; -0.51] |  | 0.10 | -7.25 | **< .001** |
| SPQ/AQ * Recall Performance | | -0.07  [-0.22; 0.08] | 0.08 | -0.93 | .351 | -0.03  [-0.18; 0.12] |  | 0.08 | -0.43 | .667 |
| Medium Predictability * SPQ / AQ * Recall Performance | | 0.04  [-0.14; 0.22] | 0.09 | 0.43 | .668 | -0.07  [-0.25; 0.11] |  | 0.09 | -0.74 | .460 |
| Low Predictability * SPQ / AQ * Recall Performance | | 0.12  [-0.07; 0.31] | 0.10 | 1.27 | .204 | 0.13  [-0.06; 0.33] |  | 0.10 | 1.38 | .168 |
| **Random Effects** | | **Variance** | | **SD** | |  | **Variance** | | **SD** | |
| Sentence | | 0.31 | | 0.56 | |  | 0.31 | | 0.56 | |
| Participant | | 0.10 | | 0.31 | |  | 0.10 | | 0.31 | |

***
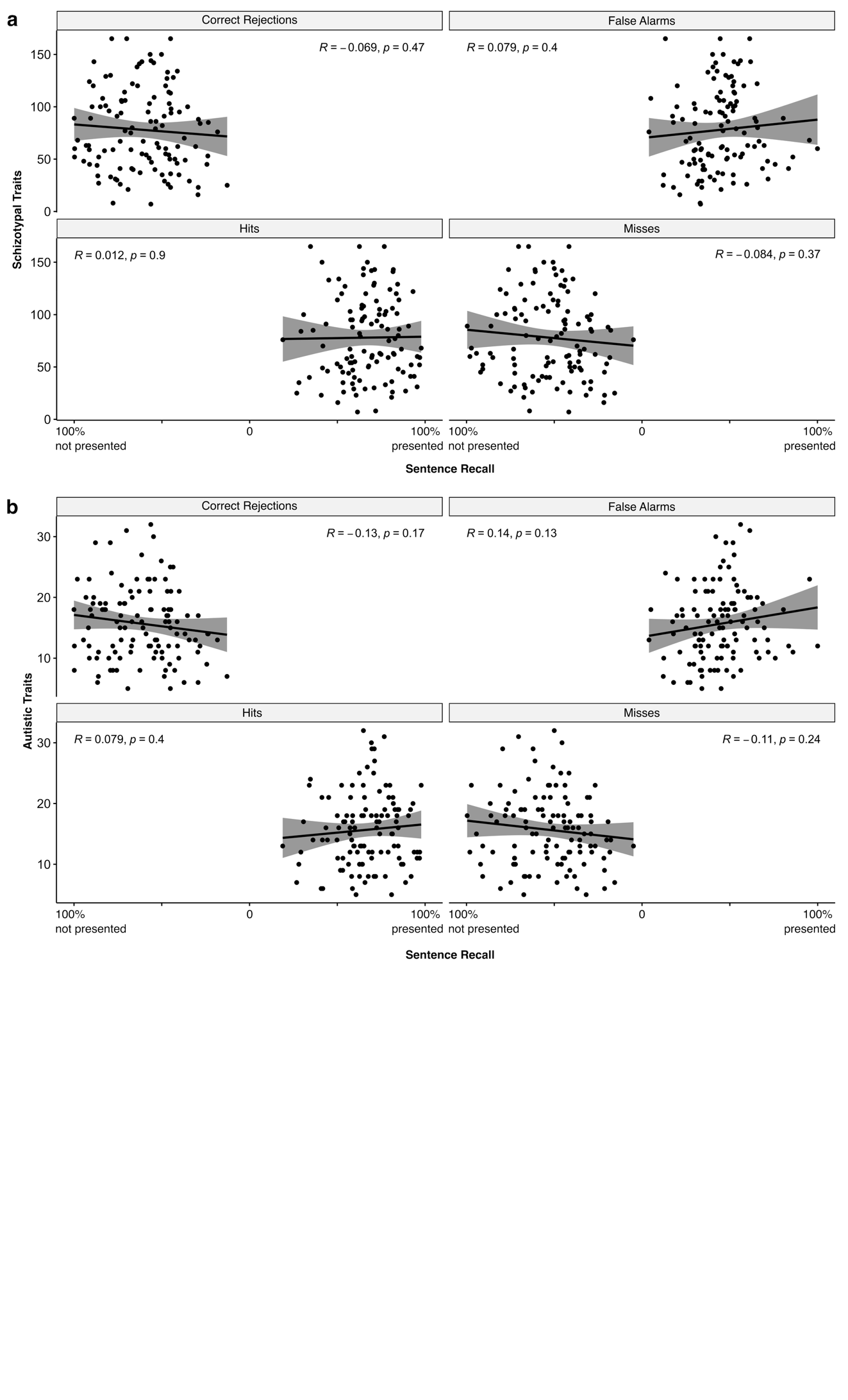
*Explorative Correlation Analyses**

***Figure S3 Correlation Analyses between Subjective Sentence Recall and Schizotypal and Autistic Traits.*** Sentence recall performance (hits, misses, correct rejections, false alarms) was defined based on the accuracy of the participants’ subjective recall rating. Pearson correlations between subjective sentence recall ratings and (a) schizotypal traits and (b) autistics traits did not reveal significant associations across the four performance categories.

*
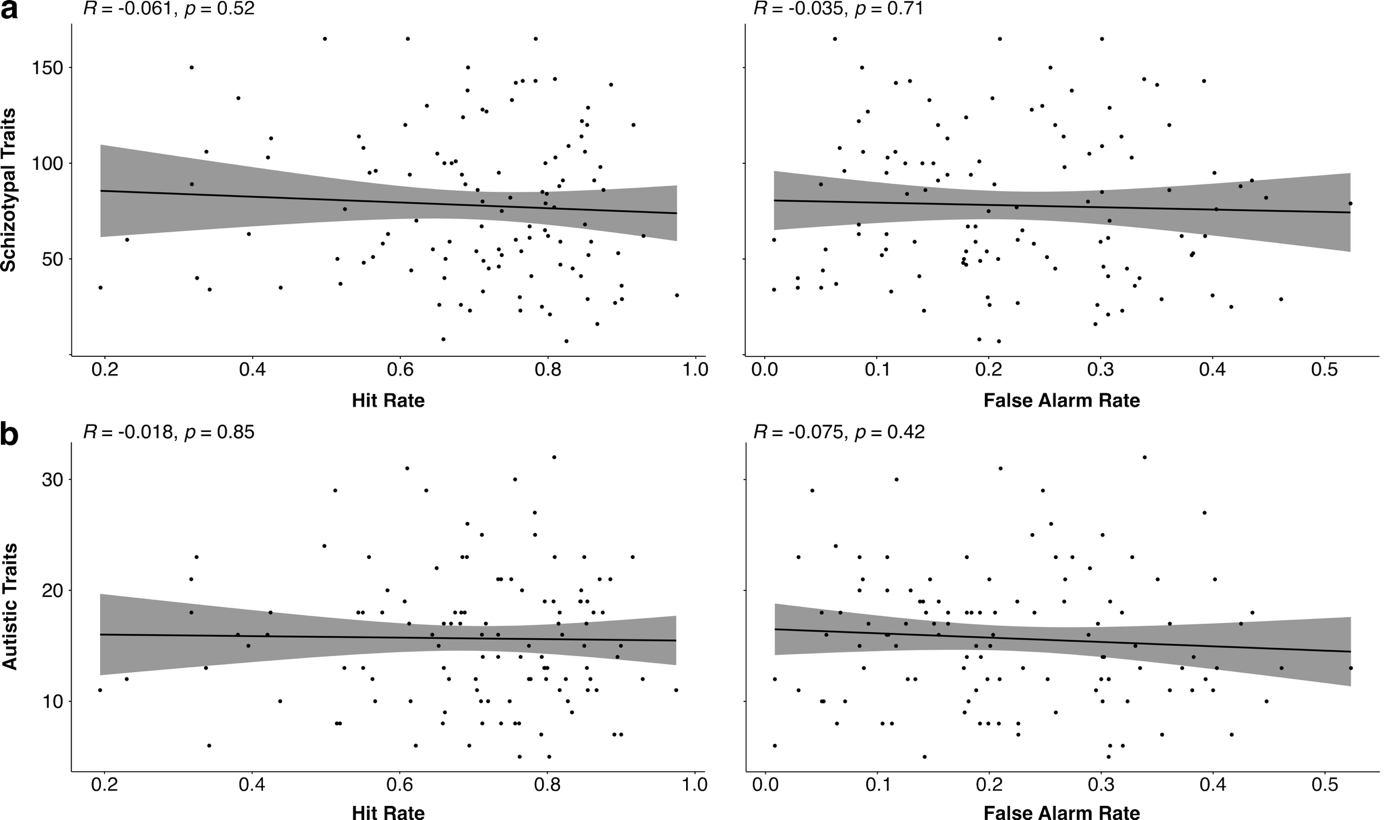
*

***Figure S4 Correlation Analyses between Hit Rates and False Alarm Rates and Schizotypal and Autistic Traits.*** Sentence recall performance (hits, misses, correct rejections, false alarms) measures were used to calculate hit rates (hits/(hits+misses) and false alarm rates (false alarms/(false alarms + correct rejections)). Pearson correlations between hit rates and false alarm rates and (a) schizotypal traits and (b) autistics traits did not reveal significant associations.

**References**

1. Baron-Cohen S, Wheelwright S, Skinner R, Martin J, Clubley E. The Autism-Spectrum Quotient (AQ): Evidence from Asperger Syndrome/High-Functioning Autism, Malesand Females, Scientists and Mathematicians. *J Autism Dev Disord*. 2001;31(1):5-17. doi:10.1023/A:1005653411471

2. Raine A. The SPQ: A Scale for the Assessment of Schizotypal Personality Based on DSM-III-R Criteria. *Schizophr Bull*. 1991;17(4):555-564. doi:10.1093/schbul/17.4.555

3. Wuthrich V, Bates TC. Confirmatory Factor Analysis of the Three-Factor Structure of the Schizotypal Personality Questionnaire and Chapman Schizotypy Scales. *J Pers Assess*. 2006;87(3):292-304. doi:10.1207/s15327752jpa8703_10

4. Klein C, Andresen B, Jahn T. Erfassung der schizotypen Persönlichkeit nach DSM-III-R: Psychometrische Eigenschaften einer autorisierten deutschsprachigen Übersetzung des “Schizotypal Personality Questionnaire” (SPQ) von Raine. [Psychometric assessment of the schizotypal personality according to DSM-III-R criteria: Psychometric properties of an authorized German translation of Raine’s “Schizotypal Personality Questionnaire” (SPQ).]. *Diagnostica*. 1997;43(4):347-369.
